# Recent Development of Mg Ion Solid Electrolyte

**DOI:** 10.3389/fchem.2020.00125

**Published:** 2020-02-25

**Authors:** Yi Zhan, Wei Zhang, Bing Lei, Hongwei Liu, Weihua Li

**Affiliations:** School of Chemical Engineering and Technology, Sun Yat-sen University, Zhuhai, China

**Keywords:** Mg batteries, solid electrolyte, phosphate, borohydride, chalcogenides, metal-organic frame (MOFs)

## Abstract

Although the successful deployment of lithium-ion batteries (LIBs) in various fields such as consumer electronics, electric vehicles and electric grid, the efforts are still ongoing to pursue the next-generation battery systems with higher energy densities. Interest has been increasing in the batteries relying on the multivalent-ions such as Mg^2+^, Zn^2+^, and Al^3+^, because of the higher volumetric energy densities than those of monovalent-ion batteries including LIBs and Na-ion batteries. Among them, magnesium batteries have attracted much attention due to the promising characteristics of Mg anode: a low redox potential (−2.356 V vs. SHE), a high volumetric energy density (3,833 mAh cm^−3^), atmospheric stability and the earth-abundance. However, the development of Mg batteries has progressed little since the first Mg-ion rechargeable battery was reported in 2000. A severe technological bottleneck concerns the organic electrolytes, which have limited compatibility with Mg anode and form an Mg-ion insulating passivation layer on the anode surface. Consequently, beneficial to the good chemical and mechanical stability, Mg-ion solid electrolyte should be a promising alternative to the liquid electrolyte. Herein, a mini review is presented to focus on the recent development of Mg-ion solid conductor. The performances and the limitations were also discussed in the review. We hope that the mini review could provide a quick grasp of the challenges in the area and inspire researchers to develop applicable solid electrolyte candidates for Mg batteries.

## Introduction

Since the commercialization proposed by Sony in 1990, Li-ion batteries (LIBs) have dominated in various fields, such as electronics, electric vehicles, and smart-grids, as the energy storage system (Goodenough and Kim, 2010; Etacheri et al., 2011; Devi et al., 2020). As the most successful battery technology nowadays, LIBs possess several advantages including high energy density, good capacity retention, no memory effect and low self-discharge, surpassing last generation of batteries i.e., lead-acid batteries, nickel metal hydride batteries (Li et al., 2020; Zeng et al., 2020). However, the growing demand for battery with even higher energy density is difficult to be satisfied by LIBs because their energy storage relies on the intercalation mechanism (Cabana et al., 2010; Choi and Aurbach, 2016; Hong et al., 2020). To further increase the energy density, great interest has been again aroused to develop lithium batteries directly using Li metal as the anode, which has remained quiescence from 1980s. The technical challenge of Li anode comes from the lithium dendrite formed on surface during cycling, which could penetrate the separator and result in the short-circuit of the battery and thus fire-catching or even explosion (Janek and Zeier, 2016; Chen et al., 2020). Despite the endeavors to suppress the dendrite formation, the progress is limited and none of the solutions meets the commercial standards (Cengiz et al., 2019; Michel et al., 2019).

Therefore, next generation advanced rechargeable batteries, such as multivalent metal (Mg, Ca, Al etc.) batteries, have aroused much interest due to high energy density in theory (Aurbach et al., 2000, 2007; Yoo et al., 2013; Wang et al., 2019). Among them, rechargeable Mg batteries are considered as the most promising battery technology because of the highest theoretical volumetric energy density of Mg anode (3,866 mAh cm^−3^), surpassing that of Li anode (2,066 mAh cm^−3^) (Aurbach et al., 2000; Bucur et al., 2015). While Li metal reacts violently with water, the reaction between Mg and water is much more stable because of the passive Mg hydroxide/oxide film formed on the surface. In addition, there is no dendrite formed for Mg anode during the reversible plating/stripping process. These advantages endow Mg anode high safety compared to Li anode (Aurbach et al., 2001). In addition, Mg is more abundant relative to Li in earth crust.

Several key challenges, however, should be overcome before the Mg battery technology comes true (Yoo et al., 2013; Saha et al., 2014; Bucur et al., 2015; Wang et al., 2020). For instance, due to the electrochemical reduction, a passivation layer is formed on Mg anode surface once Mg is on contact with conventional carbonate-based electrolyte solvents used in LIBs (Muldoon et al., 2012). While conducting for Li ion in LIBs, solid electrolyte interface (SEI) is insulating for Mg ion and thus prevents the conventional electrolyte solvents to be used in Mg batteries (Pan et al., 2020). Novel electrolytes are therefore developed for Mg batteries and most of them are based on Grignard reagents dissolved in ethereal solvents or glymes such as tetrahydrofuran (THF) (Deivanayagam et al., 2019). Nevertheless, concerns on safety and stability still remain for the high vapor pressure and the high flammability of ether-based organic solvents. Furthermore, the presence of Cl^−^ anions in Grignard reagents results in the high corrosion and the electrolytes also have narrow electrochemical operation window (<2 V vs. Mg/Mg^2+^), indicating limited practical application (Tutusaus et al., 2015). Therefore, solid-state electrolyte employed by all-solid-state Mg batteries is a safe alternative in terms of heat and mechanical shock resistance (Ikeda et al., 1987; Imanaka et al., 1999; Janek and Zeier, 2016; Famprikis et al., 2019). Nevertheless, the development of solid-state Mg ion conductor with sufficient conductivity is a key challenge at ambient temperature because of the sluggish mobility resulted by the high charge density of Mg ion (Janek and Zeier, 2016; Famprikis et al., 2019). Many efforts are thus devoted to improve the mobility of Mg ion within the solid conductor and the target is 10^−3^-10^−4^ S cm^−1^ at ambient temperature, which is comparable with those of solid electrolytes used in lithium or sodium batteries (Janek and Zeier, 2016).

In this mini review, we will present a comprehensive development of solid Mg ion conductors, including phosphates, borohydrides, metal-organic frameworks (MOFs) and chalcogenides. It highlights the performance and the limitation of each material and also discusses the conduction mechanism on the basis of the crystal structure and the strategy to improve the ionic conductivity.

## Phosphates-Based Mg ION Solid Conductors

The study on solid-state ionic conductors have aroused much interest due to the merits of high stability and good safety. It is well-accepted that the migration of ion species is quite difficult in a solid, highly affected by the valence states of the ion species. Therefore, while there are many kinds of monovalent ionic conductors with high conductivity, few choices are available for multivalent ions such as Mg^2+^ (Janek and Zeier, 2016; Famprikis et al., 2019). Na^+^ superionic conductor (NASICON) is well-known for permitting the smooth migration of Na ion species due to the well-ordered three-dimensional network structure, and therefore it is highly interested to develop NASICON-type MZr_4_(PO_4_)_6_ (M = Ca^2+^, Sr^2+^, Mg^2+^, Ba^2+^) solids as multivalent ionic conductors (Lee et al., 2019; Shao et al., 2019).

The earliest report on NASICON-type Mg^2+^ conductor came from Ikeda in 1987, who studied the system of Mg-Zr-PO_4_ in various molar ratios as Mg ion conductor using mechanochemical synthesis (Ikeda et al., 1987). Among them, MgZr_4_(PO_4_)_6_ (MZP) showed the highest conductivity of 2.9 × 10^−5^ and 6.1 × 10^−3^ S cm^−1^ at 400 and 800°C, respectively. The Tubandt's method and the electron probe microanalysis were used to confirm the charge carrier to be Mg ions. According to X-ray diffraction (XRD) measurements, the author simply claimed that the crystal structure of MZP similar to NaZr_2_(PO_4_)_3_, resulted in the best conductivity relative to analogs with other ratios, while no further evidence was provided in the report.

Imanaka and Adachi continued the exploration on NASICON-type Mg^2+^ conductor. In 1999, they intentionally varied the starting materials of MZP to be non-stoichiometric ratios so as to produce the secondary phase of Zr_2_O(PO_4_)_2_ (Imanaka et al., 1999, 2000a). With the increase of the Zr_2_O(PO_4_)_2_ content in the composite, the Mg ion conductivity increased to a maximum value of 6.92 × 10^−3^ S cm^−1^ at 800°C for Mg_1+x_Zr_4_P_6_O_24+x_+xZr_2_O(PO_4_)_2_ with x = 0.4. Too high content of Zr_2_O(PO_4_)_2_ however deteriorated the conductivity of the composite because of the insulating property of Zr_2_O(PO_4_)_2_. The presence of secondary phase was believed to enhance the relative density and thus the ionic conductivity by microscopically dispersing the Zr_2_O(PO_4_)_2_ secondary phase in the composite.

In 2001, Imanaka used the substitution strategy to improve the ionic conductivity of MZP by partially replacing Zr^4+^ with Nb^5+^ (Imanaka et al., 2000b). The substitution was used to statistically distribute mobile Mg ions to make a smooth Mg ion diffusion but it also reduced the number of migrating Mg^2+^ ion species in the solid solution electrolyte. Thus, the optimum conductivity was observed at x = 0.15 in Mg_1−2x_(Zr_1−x_Nb_x_)_4_P_6_O_24_ with 7.7 × 10^−4^ S cm^−1^ at 600°C and 3.7 × 10^−3^ S cm^−1^ at 750°C, demonstrating little enhancement compared to pristine MZP.

Later in 2016, Imanaka and Tamura claimed that most of the attempts to obtain the NASICON-type structure of Mg ion conductor were unsuccessful, because of the small ionic radius of Mg^2+^ resulting in the formation of β-Fe_2_(SO_4_)_3_-type structure at high temperature (Tamura et al., 2016). They selected HfNb(PO_4_)_3_ as the mother solid and partially substituted Hf^4+^ with Mg^2+^ to realize Mg^2+^ conduction. Although the ion conductivity of (Mg_0.1_Hf_0.9_)_4/3.8_Nb(PO_4_)_3_ at high temperature was lower than that of Mg_0.7_(Zr_0.85_Nb_0.15_)_4_(PO_4_)_6_, the Mg^2+^ ion conductivity of the former at a moderate temperature of 300°C (2.1 × 10^−6^ S cm^−1^) was 20 times higher than that of latter (1.1 × 10^−7^ S cm^−1^). The authors proposed that the good ionic conductivity was resulted from the three-dimensionally well-ordered NASICON structure and also the presence of cations with a higher valence than that of the conducting cation Mg^2+^, enabling the smooth ion migration of the latter.

Adamu and Kale proposed a sol-gel method to synthesize MZP (Adamu and Kale, 2016). The MZP synthesized in this work showed an ionic conductivity of 7.23 × 10^−3^ S cm^−1^ at 725°C. They attributed improvement in the conductivity to the sol-gel preparation route, which ensured synthesis at the molecular level and avoid the impurity produced by the solid-state route due to the inhomogeneous mixing.

Liang and Laine used spray pyrolysis method to synthesize Mg_0.5_Ce_0.2_Zr_1.8_(PO_4_)_3_ nanopowders, where Ce^4+^ partially substituted Zr^4+^ in Mg_0.5_Zr_2_(PO_4_)_3_ (Liang et al., 2018). Mg_0.5_Ce_0.2_Zr_1.8_(PO_4_)_3_ offered highest conductivity up to 3 × 10^−6^ S cm^−1^ at 280°C, which was comparable with the previous report by Imanaka.

Inspired by the fabrication of amorphous solid conductor for LIBs, such as Li_3_PO_4_, Su and Tsuruoka proposed a plasma-assisted atomic layer deposition method to fabricate amorphous oxygen-deficient Mg_2.4_P_2_O_5.4_ thin film in 2019 (Cengiz et al., 2019; Su et al., 2019). It exhibited an ionic conductivity of 1.6 × 10^−7^ S cm^−1^ at 500°C. The hopping conduction of Mg ions in the disordered amorphous phosphate matrix was believed to result in the conductivity.

Despite the effort devoted to the development of phosphate-based Mg ion conductors, the progress is very limited and the ionic conductivity of Mg solid electrolyte based on MZP is never above 10^−6^ S cm^−1^ at moderate temperature of 300°C. The aim to function well at room temperature is still far for phosphate-based ionic conductors. It seems that phosphate-based materials are not ideal candidates of solid Mg ion conductors and it is necessary to turn to novel Mg ion conductors.

## Borohydride Mg ION Solid Conductors

Since Mohtadi demonstrated the first fully inorganic and halide-free Mg electrolytes, enabling reversible Mg plating and stripping, in 2012, liquid electrolytes based on magnesium borohydride, Mg(BH_4_)_2_, have received significant attention (Mohtadi et al., 2012). Because of the reductive stability of the ${\text{BH}}_{4}^{-}$ anion, Mg(BH_4_)_2_ also arouses the interest to develop as solid Mg ion conductor. However, the conductivity of Mg(BH_4_)_2_ is very low at room temperature (10^−12^ S cm^−1^ at 30°C), resulted by the firm tetrahedral sites of ${\text{BH}}_{4}^{-}$ hindering the smooth migration of Mg ions. Proper modification of Mg(BH_4_)_2_ are thus necessary to improve the conductivity.

In 2014, Higashi first reported Mg(BH_4_)(NH_2_) as a new class of solid-state Mg ion conductor (Higashi et al., 2014). The diffusion of Mg ions in Mg(BH_4_)(NH_2_) was attributed to the Mg zigzag chain and tunneling structures in the *a* and *b* planes. It showed the ionic conductivity of 10^−6^ S cm^−1^ at 150°C and the electrochemical window of ~3 V in estimation.

Le Ruyet and Janot followed the work on Mg(BH_4_)(NH_2_) in 2019, and studied the influences of the synthesis parameters on the ionic conductivity (Le Ruyet et al., 2019). They carefully investigated the synthesis parameters and the ionic conductivity could reach as high as 3 × 10^−6^ S cm^−1^ at 100°C, which was three orders of magnitude higher than that reported by Higashi. The improvement in conductivity was attributed to the creation of a glass-ceramic-like composite due to the presence of an amorphous additional phase.

Roedern and Remhof synthesized a Mg(BH_4_)_2_ derivative by coordinating Mg^2+^ with a neutral bidentate ethylenediamine ligand to replace two BH_4_ ligand (Roedern et al., 2017). It showed a high ionic conductivity of 5 × 10^−8^ S cm^−1^ at 30°C and 6 × 10^−5^ S cm^−1^ at 70°C. The good conductivity was attributed to the partially chelated, mixed coordination of Mg^2+^ leading to its high mobility. However, the electrochemical stability of this new phase is limited to 1.2 V vs. Mg/Mg^2+^, due to the stability limitation by the ethylenediamine ligand.

Compared with phosphate-based Mg ion conductors, Mg(BH_4_)_2_ and its derives show a promising future as Mg ion conductors with high ionic conductivity at low temperature. Proper modification of Mg(BH_4_)_2_ are still necessary to improve the conductivity, stability and operating potential window.

## Chalcogenide Mg ION Solid Conductors

Chalcogenide-based materials have been developed as solid conductors for Li ion and Na ion and have shown high ionic conductivity (Ramos et al., 2018; Xuan et al., 2018; Jia et al., 2019; Wang Y. et al., 2019). It is therefore highly interesting to develop chalcogenide-based ionic conductors for Mg ion.

In 2014, Yamanaka and Tatsumisago prepared the MgS–P_2_S_5_-MgI_2_ glasses and glass-ceramics by a mechanochemical method (Yamanaka et al., 2014). The addition of MgI_2_ content in 60MgS·40P_2_S_5_ helped the formation of glass-ceramic and the conductivity monotonically increased with the increase of MgI_2_ content, showing the highest ionic conductivity to be 2.1 × 10^−7^ S cm^−1^ at 200°C. The authors proposed that the Mg_2_P_2_S_6_ crystal phase contributed to the increased conductivities. But no solid evidence was presented to support the claim in the study.

**Figure 1:**
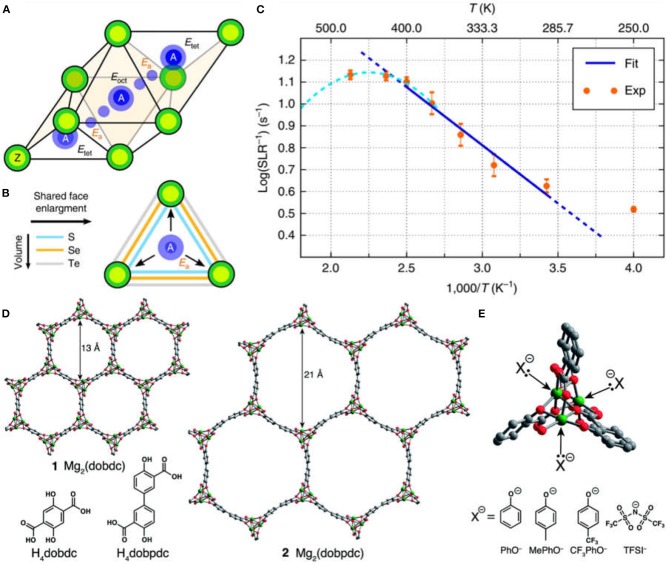
*Tet*-*oct*-*tet* migration path in the AX_2_Z_4_ framework, with energy of the *tet, oct*, and transition sites indicated by E_tet_, E_oct_, E_a_, respectively (Ea corresponds to the migration energy) **(A)**; effect of the anion size on the shared (triangular) face between *tet* and *oct* sites **(B)** and ^25^Mg static variable temperature spin lattice relaxation data collected at 7.02 T as function of temperature and Arrhenius fit **(C)**. Reproduced from Canepa et al. (2017), licensed under CC BY 4.0. structures of the metal–organic frameworks Mg_2_(dobdc) (**1**) and Mg_2_(dobpdc) (**2**), as viewed along the *c*-axis **(D)**; a close-up of the open coordination sites at the vertices of the pore that interact with nucleophilic guest species: PhO^−^ = phenolate, MePhO^−^ = 4-methylphenolate, CF_3_PhO^−^ = 4-trifluoromethylphenolate, and TFSI^−^= bis(trifluoromethanesulfonyl)imide **(E)**. Reproduced with permission from Aubrey et al. (2014), Copyright 2014, Royal Society of Chemistry.

In 2017, Canepa, Bo and Ceder demonstrated the discovery of spinel chalcogenides MgX_2_Z_4_ [X = (In, Y, Sc) and Z = (S, Se)] as a class of fast Mg ion solid conductors with the combination of theoretical and experimental studies (Canepa et al., 2017). The ambient-temperature ionic conductivity could reach as high as ~10^−4^ S cm^−1^ for MgSc_2_Se_4_ at 25°C. The fast diffusion of Mg ions was achieved by the occupation of stable Mg^2+^ site in its unfavorable tetrahedral coordination environment within spinel structure (Figures 1A,B). The measured Mg migration barrier was consistent with the computed data (Figure 1C). Such rationale could be used as a general design rule for multivalent-ion solid conductors. However, the electronic conductivity of MgSc_2_Se_4_ is ~0.04% of the ionic conductivity, which is substantially larger than other state-of-the-art alkali solid-state electrolytes (σ_e_/σ_i_~10^−4^-10^−6^%) and hinders MgSc_2_Se_4_ as an applicable solid-state conductor.

In order to make MgSc_2_Se_4_ as an applicable solid-state conductor, Wang and Fichtner tried two strategies to reduce the electronic conductivity of MgSc_2_Se_4_, including synthesizing Se-rich phase and doping with Ce^4+^ or ti^4+^ to partially substitute Sc^3+^ (Wang et al., 2019). However, neither methods could successfully reduce the electronic conductivity. The authors suggested to employ MgSc_2_Se_4_ as a surface coating to protect Mg anode or cathode materials.

A significant improvement in ionic conductivity is achieved by chalcogenide-based material, which is 10^−4^ S cm^−1^ for MgSc_2_Se_4_ at ambient temperature. Despite the promising conductivity, its high electronic conductivity hinders its application as a suitable solid conductor. Further modifications are necessary to decrease the electronic conductivity and increase the ionic conductivity at the same time.

## Metal–Organic Frameworks (MOFs) Mg ION Solid Conductors

Metal–organic frameworks (MOFs) are crystalline solids composed of metal ions coordinated by multifunctional organic molecules with a three-dimensional porous structure. The composition and structure of MOFs could be easily adjusted via the rational selection of the metal ion and organic molecule (Rouhani et al., 2019). Furthermore, they feature poor electrical conductivity and well-defined porous structure, allowing for fast ion diffusion (Zhu et al., 2019). Therefore, MOFs could be promising candidates as ideal ionic conductors for selective transport. Many MOFs have been reported as solid Li^+^ conductors, with the conductivities as high as 3 × 10^−4^ S cm^−1^ at room temperature. In contrast, the work on solid Mg conductors is still few in the field.

In 2014, Aubrey and Long first presented porous MOFs of Mg_2_(dobdc) (dobdc^4−^ = 2,5-dioxidobenzene-1,4-dicarboxylate) and its analog Mg_2_(dobpdc) (dobpdc^4−^=4,4′-dioxidobiphenyl-3,3′-dicarboxylate) as solid Mg^2+^ conductors (Aubrey et al., 2014). These MOFs exhibited ionic conductivities of up to 2.5 × 10^−4^ S cm^−1^ at room temperature after soaked in solution containing Mg salt, comparable with polymer gels. As can be seen in Figure 1D, Mg_2_(dobdc) shows the pore size of 13 Å and Mg_2_(dobpdc) has the pore size up to 21 Å, suggesting their good ability to accommodate the ion species like Mg^2+^ in high charge density. With the insertion of Mg salts, a high density of open metal sites in MOFs could capture nucleophilic anions and thus allow for the favorably free mobility of Mg ions within pores (Figure 1E). Therefore, high ionic conductivity was achieved for Mg_2_(dobpdc) impregnated with magnesium phenolates.

In 2017, Park and Dinca proposed a Cu(II)–azolate MOF (MIT-20) as a tunable solid electrolyte for Li^+^, Na^+^ and Mg^2+^ after soaking in the corresponding halide or pseudohalide salts (Park et al., 2017). Its Mg^2+^-substituted analog, MIT-20-MgBr_2_, exhibited the Mg ionic conductivity of 8.8 × 10^−7^ S cm^−1^ at the room temperature. MIT-20 was attractive for its feature of immobilizing anions by the Cu(II) metal center, allowing the favorably free migration of cations within the one-dimensional pores. Later in 2019, Miner from Dinca's group continued their work on the tunable solid electrolyte of MOF (Miner et al., 2019). The MOF was Cu_4_(ttpm)_2_·0.6CuCl_2_, possessing high surface area with plenty of Cu(II) cations to bound halide anions. Its analog, Cu_4_(ttpm)_2_·0.6CuCl_2_-MgBr_2_, exhibited an ionic conductivity of 1.3 × 10^−4^ S cm^−1^ for Mg ions, demonstrating the promising future of MOF-based solid electrolyte to optimize the ionic conductivity via the control of identity, geometry and distribution of the cation hopping sites.

Instead of pressed pellets, Luo, Tsung and Wang synthesized a Mg-MOF-74 thin film as the Mg ion conductor to eliminate interparticle gaps that were inevitable for pressed pellets and enabled studies on the inherent ionic conductivity of MOFs (Luo et al., 2019). It showed the ionic conductivity of 3.17 × 10^−6^ S cm^−1^ at the room temperature, which was in consistent with the previous report by Aubrey.

Despite the promising properties, additional salt contents in excess are required for MOFs solid conductors based on the number of available anion binding sites. Furthermore, activation energies are sometimes higher that what are expected, due to the strong pairing between cations and anions of the salts. Electrochemical stability upon cycling is another challenge for some reported MOFs-based conductors.

## Conclusions and Outlook

**Figure 2:**
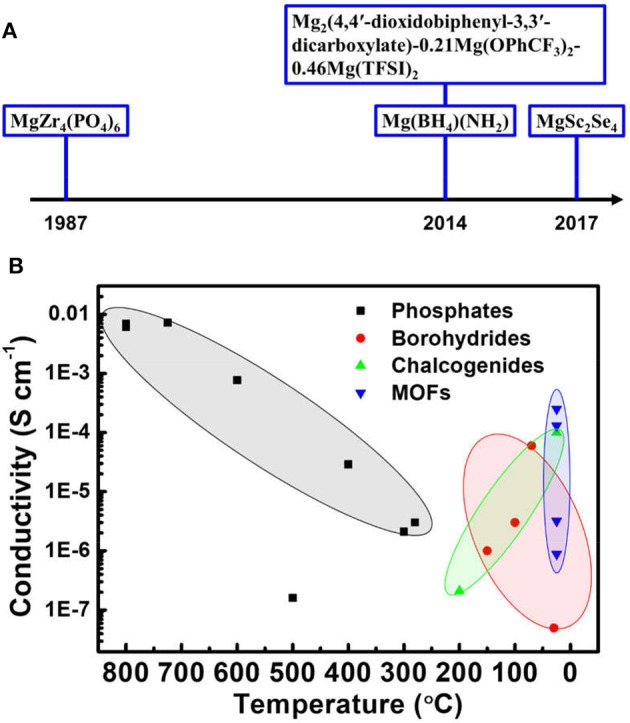
A timeline of key development of Mg ion solid conductors **(A)** and a summary of the ionic conductivities of various solid conductors as a function of temperature in the report **(B)**.

Mg battery is considered as a promising next-generation advanced battery technology possessing high theoretical volumetric energy density, good safety and high abundance. Nevertheless, before the battery technology becomes viable, several critical technological challenges should be conquered, including the development of proper electrolyte and cathodes. The SEI formed on the Mg surface is ionic-blocking for Mg ion when Mg batteries employs the conventional carbonate-based solvent commonly used in LIBs. The electrolyte development for Mg batteries therefore cannot simply mimic LIB electrolytes but requires novel design strategies. The proposal of ether-based solvent demonstrated the ability to avoid the formation of insulating passivation layer, but the practical application is still highly limited by the corrosive issues from Grignard salts and the safety concern, as well as the small electrochemical potential window. Featuring high stability and safety, ionic solid conductor could be an ideal solution to the above problems. But Mg ions have sluggish mobility within solid conductor due to the high charge density. Several types of solid conductors, including phosphates, borohydrides, chalcogenides and MOFs, have been developed to improve the performance of Mg ionic solid conductor over the recent years (Figure 2A).

As summarized in Figure 2B, the impressive progress has been achieved with the conductivity of initially 10^−3^ S cm^−1^ at 800°C improved to be 10^−4^ S cm^−1^ at ambient temperature. Despite the efforts, phosphates are still far from to meet the requirement of high ionic conductivity at low temperature and show poor progress over decades, demonstrating little promise as Mg solid conductors. In contrast, significant improvements in conductivity have been shown by the recent development of borohydrides, chalcogenides and MOFs. These novel solid conductors should be the development emphasis in next stage.

However, more work should be done before the ionic conductors are sufficiently conductive for practical consideration in Mg batteries. The electrochemical potential window should be increased to a sufficiently high level. The feasibility of the solid conductor requires the practical evaluation in combination with Mg anode and cathodes as an all-solid-state Mg battery. Furthermore, theoretical calculation could be a powerful tool to improve the performance of solid conductor.

## Author Contributions

YZ was in charge of organization and writing of the manuscript. WZ contributed the Phosphate section. BL contributed the Borohydride section. HL contributed the Chalcogenide section. WL contributed the MOFs section.

### Conflict of Interest

The authors declare that the research was conducted in the absence of any commercial or financial relationships that could be construed as a potential conflict of interest.
